# Biological ageing and frailty markers in breast cancer patients

**DOI:** 10.18632/aging.100745

**Published:** 2015-05-15

**Authors:** Barbara Brouwers, Bruna Dalmasso, Sigrid Hatse, Annouschka Laenen, Cindy Kenis, Evalien Swerts, Patrick Neven, Ann Smeets, Patrick Schöffski, Hans Wildiers

**Affiliations:** ^1^ Laboratory of Experimental Oncology (LEO), Department of Oncology, KU Leuven, and Department of General Medical Oncology, University Hospitals Leuven, Leuven Cancer Institute, Leuven, Belgium; ^2^ Department of Internal Medicine, Istituto di Ricerca a Carattere Clinico e Scientifico (IRCCS), Azienda Ospedaliera Universitaria (AOU) San Martino Istituto Nazionale Tumori (IST), Genoa, Italy; ^3^ Interuniversity Centre for Biostatistics and Statistical Bioinformatics, Leuven, Belgium; ^4^ Leuven Multidisciplinary Breast Center, University Hospitals Leuven, Belgium

**Keywords:** biomarkers, breast cancer, geriatric assessment, interleukin-6, frailty

## Abstract

Older cancer patients are a highly heterogeneous population in terms of global health and physiological reserves, and it is often difficult to determine the best treatment. Moreover, clinical tools currently used to assess global health require dedicated time and lack a standardized end score. Circulating markers of biological age and/or fitness could complement or partially substitute the existing screening tools. In this study we explored the relationship of potential ageing/frailty biomarkers with age and clinical frailty. On a population of 82 young and 162 older non-metastatic breast cancer patients, we measured mean leukocyte telomere length and plasma levels of interleukin-6 (IL-6), regulated upon activation, normal T cell expressed and secreted (RANTES), monocyte chemotactic protein 1 (MCP-1), insulin-like growth factor 1 (IGF-1). We also developed a new tool to summarize clinical frailty, designated Leuven Oncogeriatric Frailty Score (LOFS), by integrating GA results in a single, semi-continuous score. LOFS' median score was 8, on a scale from 0=frail to 10=fit. IL-6 levels were associated with chronological age in both groups and with clinical frailty in older breast cancer patients, whereas telomere length, IGF-1 and MCP-1 only correlated with age. Plasma IL-6 should be further explored as frailty biomarker in cancer patients.

## INTRODUCTION

Chronological age does not always accurately reflect functional status and life expectancy. Frail patients exhibit severely reduced physiological reserves that render them susceptible to minor stressors. More and more oncological therapies become available to older cancer patients, but an accurate evaluation of clinical frailty and general health status is crucial in treatment decision making. Geriatric assessment (GA) is currently the gold standard to evaluate the global health status and clinical frailty level of individuals [1] and is feasible in clinical practice [2], but has several drawbacks. Firstly, it is time-consuming and therefore difficult to integrate in routine clinical practice, although the use of screening tools might partly overcome this problem [3]. Secondly, although GA corresponds with important outcome measures like patient survival and toxicity of treatment, its predictive capacity is moderate, and there is certainly room for better tools. Thirdly, as it does not yield a validated ‘end score’, it is difficult to precisely quantify the patients' global health status. For this reason, some attempts to summarize and categorize GA results have been proposed in geriatric oncology such as the Balducci score, but the included elements and cut-offs are arbitrary, and do not capture the complexity of the entire ageing process (e.g. age ≥ 85 is sufficient to be categorized as ‘frail’, although it has been stated in the geriatric literature that more than half of patients above 85 are actually not frail) [4].

Several biological ageing and frailty markers described in experimental geriatrics have been proposed to reflect ‘biological age’ more accurately than clinical assessment [5], but this still needs to be proven. In fact, it is not always clear whether these biomarkers merely reflect chronological age, or rather the presence of clinical frailty. Anyhow, these ageing/frailty biomarkers have not yet filled the gap from bench to bedside to date. In addition, it should be noted that the oncogeriatric field represents a specific niche where extrapolation of general findings from geriatrics research may not be fully valid.

Telomere length represents one of the best documented markers of ageing. As telomeres were shown to represent some type of cellular ‘mitotic clock’, mean leukocyte telomere length is commonly accepted as a promising ageing biomarker. Indeed, progressive telomere attrition with increasing age has been reported repeatedly. Moreover, shorter telomeres have been linked to age-related disorders such as dementia, cardiovascular diseases, osteoporosis, chronic obstructive lung diseases, cancer, and, most importantly, to a significantly higher mortality rate in the elderly [6]. The association between telomere length and frailty or disability is however much less clear from the literature.

Plasma IL-6 levels have also been associated with mortality and/or worse outcome of several diseases, particularly cardiovascular pathology [7-9]. Several investigators have specifically linked circulating IL-6 levels to the frailty syndrome [10-13]. Notably, IL-6 rising is not an isolated phenomenon but must be seen in the context of a general age-related increase in inflammation markers, called “inflammageing” [14]. Similar age-related differences have also been described for certain inflammatory chemokines such as MCP-1 (also named CCL2, CC-chemokine ligand 2) [15-19], a protein known for its potent ability to attract and activate monocytes/macrophages. Circulating levels of RANTES, another member of the CC-chemokine subfamily, tend to change during ageing [19]. In addition to the above described molecules, certain endocrine markers also show age-related changes. More specifically, IGF-1 is inversely correlated with increasing age [20]. In mice models, disruption of the GH/IGF-1 signaling network resulting in IGF-1 reduction is associated with increase in oxidative stress in the liver, reduced lifespan, and reduced skeletal density [21]. In humans, low IGF-1 levels have been associated with frailty and decreased functionality [22] Although these candidate ageing/frailty biomarkers have been described in the geriatric field, their value in the clinical practice is far from established, and they have not formally been correlated with the frailty syndrome [23] in older cancer patients. The present study was undertaken to validate the five potential ageing/frailty markers mentioned above in a retrospective cohort of older breast cancer patients. In particular, we wanted to investigate the relationship between these markers and calendar age on the one hand, and with the different components of standard geriatric assessment on the other hand.

## RESULTS

### Patient and tumor characteristics of included subjects

In total, 244 patients were included in the analysis, of which 82 and 162 were assigned to the young and older patient groups, respectively. Median ages in the young and older groups were 40.0 years (range 27-56) and 76.0 years (range 70-90), respectively. Clinical tumor characteristics are displayed in Table 1. Descriptive statistics of all geriatric test items performed on patients from the older cohort (screening tools and geriatric assessment) are summarized in Table 2. Dependency at ADL was noted for 49.4% of patients, and 53.9% of patients showed dependency at iADL. According to Balducci's criteria, 24.1% of older patients (N=162) scored ‘fit’, 25.3% ‘vulnerable’ and 50.6% ‘frail’. Our newly developed LOFS could be calculated only for patients who completed all the tests contributing to the scoring (N=130), and the median score was 8 (Q1= 7, Q3= 9).

**Table 1:** Summary of clinical characteristics of young and old patients

Clinical characteristics	Young patients	Older patients
	N=82	N=162
Age (median in years, [IQR])	40.0 [37.0 – 44.0]	76.0 [72.0 – 80.0]
BMI (median, [IQR])	23.0 [21.3 – 25.6]	26.5 [23.9 – 29.8]
Neoadjuvant hormonal treatment (N/total, %)	8/82 (9.8%)	18/162 (11.1%)
Grade (%)		
I	14.6	15.4
II	47.6	47.5
III	37.8	36.4
unknown	0	0.6
pT^a^ (N/total, %)		
1	35/74 (47.3%)	55/144 (28.1%)
2	33/74 (44.6%)	79/144 (54.9%)
3	4/74 (5.4%)	8/144 (5.6%)
4	1/74 (1.4%)	2/144 (1.4%)
x	1/74 (1.4%)	0/144 (0%)
pN^a^ (N/total, %)		
0	44/74 (59.5%)	83/144 (57.6%)
1	15/74 (20.3%)	42/144 (29.2%)
2	9/74 (12.2%)	10/144 (6.9%)
3	6/74 (8.1%)	8/144 (5.6%)
x	0/74 (0%)	1/144 (0.7%)
Histological subtype (N/total, %)		
ductal	74/82 (90.2%)	111/162 (68.5%)
lobular	8/82 (9.8%)	25/162 (15.4%)
ductal + lobular	0/82 (0%)	2/162 (1.2%)
ductal + other	0/82 (0%)	5/162 (3.1%)
other	0/82 (0%)	19/162 (11.7%)
‘Molecular’ subtype (N/total, %)		
Lum A	49/82 (59.8%)	99/162 (61.1%)
Lum B	15/82 (18.3%)	29/162 (17.9%)
Lum B – Her2	6/82 (7.3%)	12/162 (7.4%)
Her2	4/82 (4.9%)	8/162 (4.9%)
Triple Neg	8/82 (9.8%)	14/162 (8.6%)

a Only for patients who received upfront surgery

Abbreviations: N= number of patients, IQR= interquartile range, BMI = body mass index, pT = pathological tumor size, pN = pathological lymphnodal status, Lum A= luminal A, Lum B= Luminal B, Her2= Human epidermal growth factor receptor 2 positive tumor, Triple Neg= triple negative tumor.

**Table 2:** Descriptive statistics of all geriatric test items in the older cohort

	N	%	95% CI
			
ECOG-PS (0–5)	153		
Score 0 = asymptomatic	85	55.6	47.5 – 63.6
Score 1 = symptomatic but completely ambulatory	45	29.4	22.0 – 36.8
Score 2 = symptomatic, <50% in bed during the day	4	2.6	0.02 – 5.2
Score 3 = symptomatic, >50% in bed, but not bedbound	17	11.1	6.0 – 16.2
Score 4 = bedbound	2	1.3	0 – 3.1
			
fTRST	161		
Absence of a geriatric risk profile: score 0	41	25.5	18.6 – 32.3
Presence of a geriatric risk profile: score ≥ 1	120	74.5	67.7 – 81.4
			
G8 (0–17)	141		
Absence of a geriatric profile: score >14	62	44	35.7 – 52.3
Presence of a geriatric profile: score ≤14	79	56	47.7 – 64.4
			
ADL (6–24)	162		
Independent: score 6	82	50.6	42.8 –58. 5
Dependent: score ≥ 7	80	49.4	41.5 – 57.2
			
			
iADL (0–8)	142		
Completely independent: score 8	67	47.18	38.8 – 55.6
Dependent: score <8	75	52.82	44.4 – 61.2
			
MMSE (0–30)	156		
Score ≥24 = normal cognition	142	91	86.4 – 95.6
Score 18–23 = mild cognitive decline	13	0.3	0 – 1.2
Score ≤17 = severe cognitive decline	1	0.6	0 – 1.9
			
GDS-15	156		
Score 0-4 = not at risk for depression	134	85.9	80.3 – 91.4
Score 5-15 = at risk for depression	22	14.1	8.5 – 19.7
			
MNA-SF (0–14)	141		
Normal nutritional status: score ≥12	79	56	47.7 – 64.4
Risk of malnutrition: score ≤11	62	44	35.6 – 52.3
			
MNA (0–30)	68		
Score ≥24 = normal nutritional status	21	30.9	19.7 – 42.1
Score 17 to 23.5 = risk of malnutrition	45	66.2	54.7 – 77.6
Score <17 = malnutrition	2	2.9	0 – 7.0
			
CCI (0–37)	162		
No comorbidities (score 0)	93	57.4	49.6 – 65.2
Comorbidity score 1	34	21	14.6 – 27.4
Comorbidity score ≥2	35	21.6	15.1 – 28.1
LOFS	130		
0	0	0	–
1	6	4.6	00.9 – 08.3
2	2	1.5	0 – 03.7
3	2	1.5	0 – 03.7
4	1	0.8	0 – 02.3
5	8	6.2	1.9 – 10.4
6	11	8.5	3.6 – 13.3
7	24	18.5	11.7 – 25.3
8	24	18.5	11.7 – 25.3
9	26	20	13.0 – 27.0
10	26	20	13.0 – 27.0
			
Balducci score	162		
Fit	39	24.1	17.4 – 30.8
Vulnerable	41	25.3	18.5 – 32.1
Frail	82	50.6	42.8 – 58.5

Abbreviations: N= number of patients; 95%CI = 95% confidence interval. LOFS: Leuven Oncogeriatric Frailty Score; ECOG-PS: Eastern Cooperative Oncology Group Performance status; fTRST: Flemish version of the Triage Risk Screening Tool; ADL: Activities of Daily Living; iADL: instrumental Activities of Daily Living; MMSE: Mini Mental State Evaluation; GDS: Geriatric Depression Scale; MNA: Mini Nutritional Assessment; MNA-SF: MNA-Short Form; CCI: Charlson Comorbidity Index

### Correlation of ageing biomarkers with calendar age

**Figure 1:**
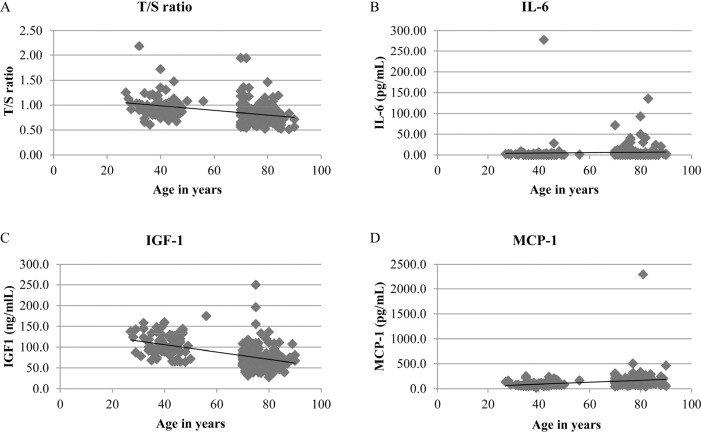
Trends of the 4 biomarkers showing significant association with chronological age (**A**) Mean telomere length (expressed as T/S ratio) versus age. N=196, Spearman correlation coefficient (r_s_) = −0.396, p<0.0001. (**B**) IL-6 versus age. N=238, r_s_ = 0.272, p<0.0001. (**C**) IGF-1 versus age. N=213, r_s_ = −0.529, p<0.001. (**D**) MCP-1 versus age. N=238, r_s_ = 0.412, p<0.0001. For graphical reasons, two outliers are not shown in both the IL-6 and MCP-1 scatterplots: IL-6 of 277.98 pg/ml in a patient aged 42 years, and MCP-1 of 2296 pg/ml in a patient aged 81 years.

Four of the measured biomarkers showed significant association with calendar age (Figure 1). The strongest association was found for circulating IGF-1 levels (Fig. 1A). The plasma biomarkers IL-6 and MCP-1 also showed significant age-related changes, whereas no significant relationship with age could be demonstrated for RANTES. Mean leukocyte telomere length, measured as T/S ratio (which could be measured in 76 young and 120 old patients), significantly decreased with increasing age.

### Relation between ageing biomarkers and clinical markers of frailty

**Figure 2:**
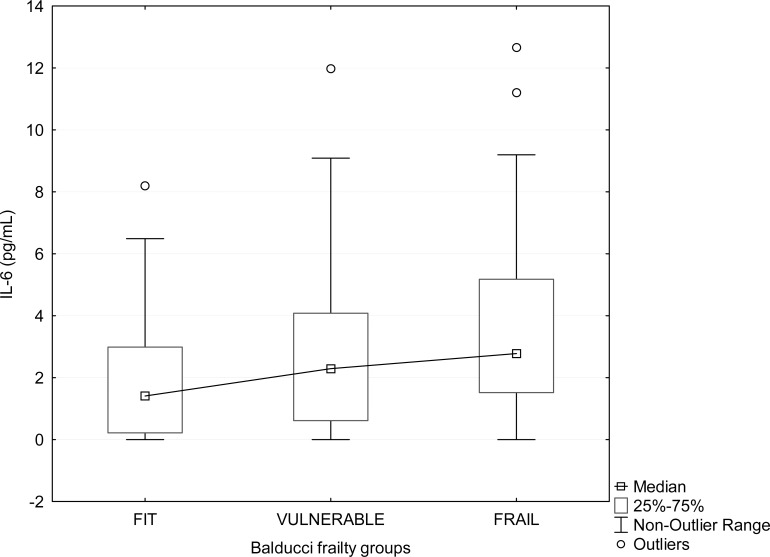
Boxplot showing the relation between plasma IL-6 and frailty status determined by Balducci Frailty Score. Frailty groups according to Balducci's test are displayed on the X axis. In each group, some extreme values are not shown for graphical reasons (2 values in ‘fit’, 2 values in ‘vulnerable’, and 8 values in ‘frail’ group).

First, we correlated the different biomarkers with frailty level according to the Balducci score. We found no difference in mean leukocyte telomere length between ‘fit’, ‘vulnerable’ and ‘frail’ patients (N=120), median T/S ratios being 0.6, 0.7 and 0.7, respectively (p = 0.391). Likewise, IGF-1, RANTES and MCP-1 plasma levels did not show any correlation with Balducci category (all p>0.4). In contrast, IL-6 plasma levels were significantly different between the 3 Balducci categories (N= 158): median values for fit, vulnerable and frail subjects were 1.4, 2.3 and 2.8 pg/ml, respectively (p = 0.019). Box plots are shown in Figure 2.

Next, we studied the relationship between the different biomarkers and LOFS, and made similar observations. No association was found between LOFS and telomere length, IGF-1, RANTES or MCP-1. For IL-6 however, a significant correlation with LOFS was observed (p=0.0131) (Table 3).

**Table 3:** Spearman correlations between IL-6 and continuous geriatric assessment scores in the old cohort

GA scores	IL-6		
	N	r_s_	p
LOFS	129	−0.218	0.0131
ECOG	149	0.244	0.0028
fTRST	157	0.078	0.3288
G8	137	−0.129	0.1320
ADL24	145	0.205	0.0134
IADL8	141	−0.202	0.0163
MMSE	152	−0.093	0.2525
GDS_15	152	0.028	0.7329
MNA-SF	137	−0.118	0.1691
MNA30	65	−0.368	0.0026
CCI	158	0.154	0.0539

Abbreviations: N = number of patients, r_s_ = Spearman correlation coefficient, p= p value. LOFS: Leuven Oncogeriatric Frailty Score; ECOG-PS: Eastern Cooperative Oncology Group Performance status; fTRST: Flemish version of the Triage Risk Screening Tool; G8: /; ADL: Activities of Daily Living; iADL: instrumental Activities of Daily Living; MMSE: Mini Mental State Evaluation; GDS: Geriatric Depression Scale; MNA-SF: Mini Nutritional Assessment-Short Form; CCI: Charlson Comorbidity Index

Lastly, we evaluated possible correlations of the different biomarkers with all the separate GA items that are evaluated on a semi-continuous scoring scale: ECOG-PS, fTRST, G8, ADL, iADL, MMSE, GDS15, MNA-SF and CCI. Neither telomere length, nor RANTES and MCP-1 correlated with any of the above mentioned items (all p>0.070, data not shown). Circulating levels of IGF-1 were inversely correlated with CCI (N=133; Spearman correlation coefficient= −0.195, p= 0.0248) (Supplementary Table 2) but not with any of the other GA parameters. The most convincing results were obtained for IL-6 (Table 3): there was a significant correlation with ECOG-PS, ADL, iADL, and a borderline significant correlation with the CCI (p=0.0539). IL-6 levels were, however, not associated with MMSE, MNA-SF, nor with any of the geriatric screening tools (fTRST and G8). Furthermore, we examined if IL-6 levels were associated with the incidence of falls during the past year. Although patients giving a positive answer to this question showed slightly increased IL-6 levels (median IL-6 value 2.7 pg/ml versus 2.3 pg/ml for patients giving a negative response), the difference was not statistically significant (p=0.154). Similarly, none of the other biomarkers correlated with falls.

### Correlations between the different ageing biomarkers

We also evaluated possible relationships between the different ageing biomarkers. Table 4 shows that, except for RANTES, all the biomarkers (telomere length, IL-6, MCP-1, IGF-1) correlated with each other to a lesser or higher extent.

**Table 4:** Correlations between biomarkers in the entire cohort (young and older patients)

Biomarker 1	Biomarker 2	Spearman's correlation coefficient	p-value
Telomere length	IL-6	−0.15553	0.0317
	MCP-1	−0.13009	0.0721
	RANTES	−0.04132	0.5693
	IGF-1	0.25608	0.0006
IL-6	MCP-1	0.22944	0.0004
	RANTES	0.02476	0.7051
	IGF-1	−0.24374	0.0003
MCP-1	RANTES	0.01760	0.7871
	IGF-1	−0.34022	<.0001
RANTES	IGF-1	0.07644	0.2667

### Correlations between ageing biomarkers and tumor characteristics

No significant association was found between any of the ageing biomarkers and tumor size (pT)[29], nodal status (pN), histologic breast carcinoma subtype (ductal, lobular, combined or other) or molecular tumor subtype (luminal A, luminal B/Her2-, luminal B/Her2+, non-luminal Her2+, triple-negative) (data not shown).

## DISCUSSION

In this study we have investigated the associations between calendar age and clinical ageing/frailty on the one hand, and a panel of biological markers, measurable in blood, on the other hand. In addition, we also describe a novel approach to compile the results of the different geriatric assessment test items into a single output score on a 10-points scale.

Geriatric assessment is currently the best way to assess the level of fitness in oncogeriatric patients, in order to plan an adequate therapeutic strategy. It allows to detect unknown geriatric problems and to adapt treatment regimens accordingly [2, 30-32]. GA consists of different questionnaires and tests that have been validated independently, each reflecting a specific aspect of the geriatric phenotype. However, interpreting GA results and translating them into a risk profile is challenging, since no real ‘end score’ exists. Several frailty models have been developed in general geriatrics, but Balducci and Extermann were the first to suggest a classification of cancer patients into 3 groups (‘fit’, ‘vulnerable’ and ‘frail’) depending on their GA result [33], with the purpose of guiding treatment decisions and predict life expectancy. For instance, frail patients would solely be administered palliative treatments, while a specific individualized approach (e.g. dose reduction at the start with subsequent escalation) would be applied for vulnerable patients. Considering the complexity of ageing, and the wide variety of aspects that are evaluated by GA, the Balducci classification is a simplified tool with many shortcomings and was probably meant as a starting point for future refinement. We have developed a new method, designated LOFS, to calculate a global GA end score that integrates 5 fundamental aspects determining a patient's fitness/frailty status, i.e. capability to autonomously perform activities of daily living (ADL and iADL), mental state (MMSE), nutritional state (MNA-SF) and comorbidities (CCI). A direct comparison between Balducci score and LOFS was not possible, as there is no gold standard to compare them to. However, LOFS is more refined than the Balducci score, as its semi-continuous scoring system is in line with recent evolution in the geriatric research field, where frailty is more and more seen as a continuous event (cumulative deficit model) [4]. In keeping with the Balducci criteria, a patient can be categorized as ‘frail’ solely based on advanced age (85 years or older), or sporadic incontinence. However, patients fulfilling these criteria are not necessarily frail individuals with a global health status being too poor to tolerate cancer chemotherapy. Undertreatment, due to fear of toxicity or intolerance, is a frequent problem in older breast cancer patients and results in an increased risk of relapse [34]. The value of LOFS in predicting toxicity of treatment and general outcome (survival) of patients will be comprehensively assessed in future studies (we are currently conducting a large survival study in a separate cohort).

As GA alone is not sufficient to predict outcome and tolerance to chemotherapy[35, 36], there is an increasing interest to integrate it with specific biomarkers that reflect biological ageing and frailty in individuals affected by cancer, in order to get a more complete picture of the patients' physiological reserves and capability to tolerate chemotherapy. Despite the well-documented correlation of several measurable molecules with age/frailty in the general (non-cancer) population [9-13, 15, 17, 18, 20, 22], none of these markers has emerged as “the” ageing biomarker of choice. Even less information is available on ageing biomarkers that could be valuable in oncogeriatric patients. This is partly due to complexity arising from the extent of the malignant disease, mechanisms of ageing and possible interactions between these two processes. We have explored several candidate ageing biomarkers in our breast cancer study cohort, and examined their relation to the patient's frailty status, either according to Balducci or our newly developed LOFS. Our results show that the biomarkers correlate at least as good with age/frailty defined according to LOFS, as Balducci. In our patient population, plasma IL-6 emerged as the strongest frailty marker. It was not only associated with Balducci category and LOFS, but also with other items of the GA not included in the calculation of these global scores, like for instance ECOG-PS. A correlation between plasma IL-6, ageing/frailty and even with mortality had previously been consistently observed in numerous studies on general (non-cancer) geriatric populations [7-13, 37]. We have shown that plasma IL-6 also correlates with calendar age. Age-related rising of circulating IL-6 is believed to originate from ageing of the immune system, generally referred to as ‘immunosenescence’. This results in an altered profile of circulating leukocytes, but also provokes an imbalance of pro- and anti-inflammatory cytokines and chemokines. This ageing-related pro-inflammatory status, called ‘inflammageing’, has been correlated with dementia, Parkinson's disease, atherosclerosis, type 2 diabetes, sarcopenia, functional disability and death [14, 38]. We now demonstrated that circulating IL-6 levels are associated with both chronological age and frailty in a selected breast cancer population. The additional predictive value of IL-6, supplementary to that of clinical frailty assessment, for outcome and treatment toxicity in (breast) cancer patients should be explored in the future.

For plasma MCP-1 we found a strong correlation with calendar age but no association with clinical frailty. Although association of MCP-1 with atherosclerosis has been documented, circulating MCP-1 levels seem to be primarily correlated with chronological age [15-19]. In line with these earlier reports, our results suggest that MCP-1 merely reflects chronological age, which does not always accurately mirror the patient's physiological reserves and functional status. The lack of association with clinical frailty does not make MCP-1 an attractive biomarker to be used in the clinic for treatment decisions. Data on the chemokine RANTES/CCL5 are even less consistent: RANTES has been proposed as an ageing biomarker because its plasma concentrations have been shown to increase with ageing [39], but in our study we did not find any association with calendar age nor with clinical frailty.

As expected, mean leukocyte telomere length inversely correlated with chronological age in our study population. However, we did not detect any correlation between mean telomere length in circulating white blood cells and functional status. Shorter telomeres have been linked to age related diseases, a higher mortality rate [6], and also to premature ageing syndromes like progeria or trichotiodystrophy [40, 41]. However, studies investigating the relationship between telomere length and frailty/disability showed conflicting results [42-48]. It has been suggested that biomarker associations with health outcomes may differ between very old and younger old populations (through a positive selection effect or survivor effect) [6, 45, 49]. Our population did not contain an overload of ‘very old’, as the median age was 76 years and only 16% of patients were above 80. Nevertheless, we did not find a significant relationship between telomeres and frailty. It should be kept in mind, though, that the oncogeriatric population is probably a biased one, as severely frail older cancer patients will rarely be referred to the oncologists for specialist care. In this regard, observations made on general geriatric populations might not be valid in oncogeriatric populations.

To our knowledge, the association between functional status and mean leukocyte telomere length has never been investigated in older cancer patients; our study is the first to report the lack of such correlation in this specific setting.

The relevance of the IGF-1 pathway in mammalian longevity was initially demonstrated in rodents (calorie restriction was shown to decrease IGF-1 levels with increased lifespan as result) and knockout mice [50]. In humans, low IGF-1 levels have been shown to inversely correlate with increasing age [20], and some studies suggest a link with frailty/functionality [22, 51]. In our study population, IGF-1 showed no association with GA components, except for a significant correlation with CCI. Yet, compared to the other investigated molecules, IGF-1 showed the strongest association with calendar age. Thus, like plasma MCP-1, circulating IGF-1 seems to be linked with the chronological age but not with ageing-related functional decline and frailty.

In conclusion, we have investigated the relationship between several potential biomarkers of ageing/frailty, and the different components of the GA, within a specific breast cancer population. We confirmed IL-6 as a promising marker in predicting frailty, besides its correlation with age. MCP-1, IGF-1 and leukocyte telomere length were correlated with chronological age, but not with frailty, and apparently reflect only the time aspect of ageing, but not the possible functional consequences (i.e. clinical frailty) of the ageing process. However, lack of correlation with frailty status at the time of diagnosis does not necessarily mean that these markers have no value in guiding treatment choices. Hence, the next steps to be undertaken are prospective validation of these and perhaps other markers like for instance circulating microRNAs, in predicting outcome including survival and short- and long term toxicity from different treatment modalities. We are currently conducting an extensive prospective ageing biomarker study in breast cancer patients receiving chemotherapy. The ultimate goal would be the identification of robust frailty biomarkers that can add on, or maybe even (partly) replace, the extensive clinical GA that is suggested nowadays.

We also designed the LOFS, a novel comprehensive method to categorize patients on the basis of their GA results. This new 10-point scoring system reflects the general condition of a patient in a more refined way and allows a more subtle interpretation of the GA results taking into account 5 crucial domains of GA, while at the same time retaining the simplicity of a single end score that is desirable for application in daily practice.

The field of medicine in general, but especially geriatric oncology, is evolving more and more towards a patient-tailored approach in which accurate frailty assessment instruments, such as easy-to-measure biomarkers and reliable but simple clinical evaluation tools, could be very helpful.

## MATERIALS AND METHODS

### Patient selection

#### 1. Selection of the older patients group

From 2004 on, several prospective projects integrating geriatric assessment in older cancer patients were performed in our center (University Hospitals Leuven, Leuven, Belgium) and all results obtained throughout the years were gathered in a GA database. From this database, all patients aged ≥ 70 years with new diagnosis of early or locally advanced (i.e. non-metastatic), primary or second primary breast cancer with GA performed before initiation of any chemotherapy, radiotherapy or surgery, were retrospectively selected. Neo-adjuvant antihormonal treatment was allowed between the time of diagnosis and the time of GA, since we expected virtually no, or only very minor impact on the result of GA. From this primary selection, we chose patients for whom a blood sample collected at diagnosis (i.e. before administration of any treatment or performance of any surgical procedure) was available from the large-scale breast cancer blood bank that was established at our hospital from 2003 onwards by the Leuven Multidisciplinary Breast Center (LMBC), and that contains blood specimens from more than 4000 breast cancer patients. In total, 162 patients fulfilled all these inclusion criteria.

#### 2. Selection of the young patients group

A second group, consisting of younger breast cancer patients, was selected from the LMBC database and biobank. Inclusion was based on the same criteria as described above, except for age and GA. We aimed for a broad age range below the cut-off of 60 years. From this group, a final selection of 82 patients was made so as to ensure that both cohorts (older versus young) contained similar percentages of Luminal A-like, Luminal B-like, Luminal B-HER2 positive, HER2 positive and Triple Negative breast tumors, as defined in one of our previous publications [24].

### Collection of plasma and leukocyte DNA

Blood sampling and isolation of plasma (collection started in 2003) and DNA (collection started in 2007) for experimental use is routinely performed in our hospital, in all new breast cancer patients who give written informed consent for the LMBC biobanking project. The procedures were previously described by Hatse S. et al. [25].

### Measurements of cytokine/chemokine levels in plasma

IL-6 was measured using LEGEND MAX^TM^ ELISA kit (BioLegend). The analysis for CCL5/RANTES, CCL2/MCP-1, and IGF-1 was carried out with Quantikine ELISA kit (R&D Systems). All procedures were performed following the manufacturers' instructions. Read-out was performed by dual spectrophotometric measurement: absorbance measured at 570 nm was subtracted from absorbance measured at 450 nm. All samples were assayed in duplicate. On each microplate, a standard curve, obtained from dilution of a standard with known concentration, was included. Concentrations of samples were calculated from the standard curve using a logistic curve-fitting algorithm.

### Mean leukocyte telomere length

Mean leukocyte telomere length was measured for all patients with a leukocyte DNA sample collected at diagnosis (i.e. patients diagnosed in 2007 or later). Every DNA sample was first tested for DNA fragmentation by electrophoresis on a 1% agarose gel. Fragmented DNA samples were excluded from further analysis. Telomere length was determined using the qPCR-based method developed by R. Cawthon [26]. Briefly, the relative amount of telomeric DNA (“T/S ratio”) is calculated based on the Cp values obtained for telomeric DNA (“T”) and for the single-copy housekeeping gene 36B4 (“S”), measured in the same sample. All samples were assayed twice in independent qPCR runs, each time in triplicate wells. Each run included a dilution series (i.e. 80, 20, 5 and 1.25 ng) of human standard DNA (Human Genomic DNA, Roche). The “T/S ratio” for an experimental sample is the amount (ng) of standard DNA that matches the experimental sample for copy number of the telomere template (“T”), divided by the amount (ng) of standard DNA that matches the experimental sample for copy number of the single-copy gene (“S”) Primer pairs used were 5′-ACACTAAGGTTTGGGTTTGGGTTTGGGTTTGGGTTAGTGT-3′ and 5′-TGTTAGGTATCCCTATCCCTATCCCTATCCCTATCCCTAACA-3′ for telomeres and 5′-CAGCAAGTGGGAAGGTGTAATCC-3′ and 5′-CCCATTCTATCATCAACGGGTACAA-3′ for 36B4. The reaction mixture contained 1x LightCycler 480 SYBR Green I Master (Roche, Indianapolis, US), telomere or 36B4 forward and reverse primers at 0.6 μM (telomeres)or 0.5 μM (36B4) each, and 20 ng of template DNA in a total volume of 20 μL. Plates were run on a Roche LightCycler 480 platform, using the following thermal cycling program: activation for 10 min at 95°C; two initiation cycles of 15s at 95°C followed by 15s at 49°C; 35 amplification cycles of 15s at 95°C, 10s at 60°C and 15s at 72°C. Thereafter, melting curves were also established to check amplicon purity.

### Geriatric assessment

Patient scores at different tests, included in the GA, were available from our GA database. The following items were mostly available: the screening tools G8 and Flemish version of the Triage Risk Screening Tool (fTRST), Eastern Cooperative Oncology Group Performance Status (ECOG-PS), functional status measured by Activities of Daily Living (ADL) and instrumental Activities of Daily Living (iADL), Geriatric Depression Scale (GDS-15), Mini Mental Evaluation-30, and Mini Nutritional Assessment- 14 items (MNA-SF). Since the geriatric assessments performed in the different oncogeriatric projects constituting our GA database were not exactly identical, there were rare missing cases for some of the scales, but for the majority, all scale results were available. Charlson Comorbidity Index (CCI) at the time of diagnosis was calculated retrospectively for each patient using the electronic patient files. More details on the scales and references can be found in another recently published paper from our group [2].

### Balducci score

A level of frailty was assigned to each patient using the criteria suggested by Balducci and Extermann [27, 28]. ‘fit’ was assigned to patients without ADL or iADL impairment (i.e. patients independent at all items), and with no or only mild comorbidities; ‘vulnerable’ was assigned to patients with dependency at 1 or more iADL items, and/or with 1 or 2 severe comorbidities; ‘frail’ was assigned to patients ≥85 years of age, or patients dependent at 1 or more ADL items, and/or exhibiting 3 or more severe comorbidities. Patients with documentation of one or more geriatric syndromes (dementia, falls, delirium, depression, incontinence, osteoporosis, neglect and abuse, failure to thrive) were also categorized as ‘frail’.

### Leuven Oncogeriatric Frailty Score (LOFS)

In the geriatrics world, frailty is more and more seen as a cumulative deficit disorder, and should thus be appraised as a continuous spectrum, rather than a dichotomized or trichotomized status[4]. Therefore, we developed the LOFS, a semi-continuous frailty score, based on internationally validated cut-offs for ADL, iADL, MMSE, MNA-SF and CCI. A detailed overview of the score compilation is shown in Supplementary Table 1. The scoring range for each separate test is trichotomized, the lowest part (worst score range for this particular test) resulting in a LOFS +0 (no contribution to the final 10-points LOFS score), the middle part in +1 (contribution of 1 point to the final score), and the highest part in +2 (contribution of 2 points). Contributions from the 5 tests are added up to result in a total score on a scale from 0 (poorest score; patient suffering from extreme frailty) to 10 (best score, fit patient). Individual results from the LOFS should be interpreted as a gradation of severity in the spectrum of frailty between both extremes.

### Statistical analysis

We analyzed correlations between ageing biomarkers and calendar age, and between ageing biomarkers and clinical frailty (defined as by Balducci test or LOFS). In addition, we also studied correlations of ageing biomarkers with each of the individual geriatric assessment tools separately and correlations among the different ageing biomarkers. Since distinct breast tumor subtypes might have a different impact on the host and tumor stroma (e.g. triple negative subtype are associated with stronger immune response), we also studied the influence of tumor subtype, tumor stage (pT) and nodal status (pN) on the biomarkers.

Associations between continuous and discrete variables were evaluated using the Mann-Whitney U test (for two levels) or the Kruskal-Wallis test (for more than two levels). Associations between two continuous variables, or between a continuous and an ordinal categorical variable, were analyzed by the Spearman correlation coefficient. Associations between two discrete variables were analyzed using the Fisher exact test.

The cut-off for statistical significance was set at p= 0.05.

### Ethical aspects

The LMBC biobank project and this study have been approved by the Ethics Committee of the University Hospitals Leuven.

## SUPPLEMENTARY INFORMATION, TABLES AND FIGURES



## References

[1] Wildiers H, Heeren P, Puts M (2014). International Society of Geriatric Oncology Consensus on Geriatric Assessment in Older Patients With Cancer. J Clin Oncol.

[2] Kenis C, Bron D, Libert Y (2013). Relevance of a systematic geriatric screening and assessment in older patients with cancer: results of a prospective multicentric study. Ann Oncol.

[3] Decoster L, Van Puyvelde K, Mohile S (2015). Screening tools for multidimensional health problems warranting a geriatric assessment in older cancer patients: an update on SIOG recommendationsdagger. Ann Oncol.

[4] Clegg A, Young J, Iliffe S (2013). Frailty in elderly people. Lancet.

[5] Pallis AG, Hatse S, Brouwers B (2014). Evaluating the physiological reserves of older patients with cancer: the value of potential biomarkers of aging?. J Geriatr Oncol.

[6] Cawthon RM, Smith KR, O'Brien E (2003). Association between telomere length in blood and mortality in people aged 60 years or older. Lancet.

[7] Harris TB, Ferrucci L, Tracy RP (1999). Associations of elevated interleukin-6 and C-reactive protein levels with mortality in the elderly. Am J Med.

[8] Haugen E, Gan LM, Isic A (2008). Increased interleukin-6 but not tumour necrosis factor-alpha predicts mortality in the population of elderly heart failure patients. Exp Clin Cardiol.

[9] Giovannini S, Onder G, Liperoti R (2011). Interleukin-6, C-reactive protein, and tumor necrosis factor-alpha as predictors of mortality in frail, community-living elderly individuals. J Am Geriatr Soc.

[10] Cohen HJ, Pieper CF, Harris T (1997). The association of plasma IL-6 levels with functional disability in community-dwelling elderly. J Gerontol A Biol Sci Med Sci.

[11] Ershler WB, Keller ET (2000). Age-associated increased interleukin-6 gene expression, late-life diseases, and frailty. Annu Rev Med.

[12] Leng SX, Xue QL, Tian J (2007). Inflammation and frailty in older women. J Am Geriatr Soc.

[13] Ferrucci L, Harris TB, Guralnik JM (1999). Serum IL-6 level and the development of disability in older persons. J Am Geriatr Soc.

[14] De Martinis M, Franceschi C, Monti D, Ginaldi L (2005). Inflamm-ageing and lifelong antigenic load as major determinants of ageing rate and longevity. FEBS Lett.

[15] Inadera H, Egashira K, Takemoto M (1999). Increase in circulating levels of monocyte chemoattractant protein-1 with aging. J Interferon Cytokine Res.

[16] Deo R, Khera A, McGuire DK (2004). Association among plasma levels of monocyte chemoattractant protein-1, traditional cardiovascular risk factors, and subclinical atherosclerosis. J Am Coll Cardiol.

[17] Antonelli A, Rotondi M, Fallahi P (2006). Increase of CXC chemokine CXCL10 and CC chemokine CCL2 serum levels in normal ageing. Cytokine.

[18] Mariani E, Cattini L, Neri S (2006). Simultaneous evaluation of circulating chemokine and cytokine profiles in elderly subjects by multiplex technology: relationship with zinc status. Biogerontology.

[19] Gerli R, Monti D, Bistoni O (2000). Chemokines, sTNF-Rs and sCD30 serum levels in healthy aged people and centenarians. Mech Ageing Dev.

[20] Barzilai N, Huffman DM, Muzumdar RH, Bartke A (2012). The critical role of metabolic pathways in aging. Diabetes.

[21] Gong Z, Kennedy O, Sun H (2014). Reductions in serum IGF-1 during aging impair health span. Aging Cell.

[22] Leng SX, Cappola AR, Andersen RE (2004). Serum levels of insulin-like growth factor-I (IGF-I) and dehydroepiandrosterone sulfate (DHEA-S), and their relationships with serum interleukin-6, in the geriatric syndrome of frailty. Aging Clin Exp Res.

[23] Mohler MJ, Fain MJ, Wertheimer AM (2014). The Frailty syndrome: clinical measurements and basic underpinnings in humans and animals. Exp Gerontol.

[24] Brouckaert O, Schoneveld A, Truyers C (2013). Breast cancer phenotype, nodal status and palpability may be useful in the detection of overdiagnosed screening-detected breast cancers. Ann Oncol.

[25] Hatse S, Lambrechts D, Verstuyf A (2012). Vitamin D status at breast cancer diagnosis: correlation with tumor characteristics, disease outcome, and genetic determinants of vitamin D insufficiency. Carcinogenesis.

[26] Cawthon RM (2009). Telomere length measurement by a novel monochrome multiplex quantitative PCR method. Nucleic Acids Res.

[27] Balducci L, Extermann M (2000). Management of the frail person with advanced cancer. Crit Rev Oncol Hematol.

[28] Balducci L, Extermann M (2000). Management of cancer in the older person: a practical approach. Oncologist.

[29] Greene FL, Sobin LH, Gospodarowicz MK, Wittekind V (2009). Breast tumours. TNM classification of malignant tumours.

[30] Wildiers HKC (2012). Comprehensive geriatric assessment (CGA) in older oncological patients: Why and how?. Journal of Geriatric Oncology.

[31] Maas HA, Janssen-Heijnen ML, Olde Rikkert MG, Machteld Wymenga AN (2007). Comprehensive geriatric assessment and its clinical impact in oncology. Eur J Cancer.

[32] Wedding U, Kodding D, Pientka L (2007). Physicians' judgement and comprehensive geriatric assessment (CGA) select different patients as fit for chemotherapy. Crit Rev Oncol Hematol.

[33] Winograd CH, Gerety MB, Chung M (1991). Screening for frailty: criteria and predictors of outcomes. J Am Geriatr Soc.

[34] Bouchardy C, Rapiti E, Fioretta G (2003). Undertreatment strongly decreases prognosis of breast cancer in elderly women. J Clin Oncol.

[35] Extermann M, Boler I, Reich RR (2012). Predicting the risk of chemotherapy toxicity in older patients: the Chemotherapy Risk Assessment Scale for High-Age Patients (CRASH) score. Cancer.

[36] Hurria A, Togawa K, Mohile SG (2011). Predicting chemotherapy toxicity in older adults with cancer: a prospective multicenter study. J Clin Oncol.

[37] Jenny NS, French B, Arnold AM (2012). Long-term assessment of inflammation and healthy aging in late life: the Cardiovascular Health Study All Stars. J Gerontol A Biol Sci Med Sci.

[38] Franceschi C, Capri M, Monti D (2007). Inflammaging and anti-inflammaging: a systemic perspective on aging and longevity emerged from studies in humans. Mech Ageing Dev.

[39] Mansfield AS, Nevala WK, Dronca RS (2012). Normal ageing is associated with an increase in Th2 cells, MCP-1 (CCL1) and RANTES (CCL5), with differences in sCD40L and PDGF-AA between sexes. Clin Exp Immunol.

[40] Burtner CR, Kennedy BK (2010). Progeria syndromes and ageing: what is the connection?. Nat Rev Mol Cell Biol.

[41] Hoeijmakers JH (2009). DNA damage, aging, and cancer. N Engl J Med.

[42] Woo J, Tang NL, Suen E (2008). Telomeres and frailty. Mech Ageing Dev.

[43] Mitnitski AB, Graham JE, Mogilner AJ, Rockwood K (2002). Frailty, fitness and late-life mortality in relation to chronological and biological age. BMC Geriatr.

[44] Mitnitski AB, Mogilner AJ, Rockwood K (2001). Accumulation of deficits as a proxy measure of aging. ScientificWorldJournal.

[45] Martin-Ruiz CM, Gussekloo J, van Heemst D (2005). Telomere length in white blood cells is not associated with morbidity or mortality in the oldest old: a population-based study. Aging Cell.

[46] Harris SE, Deary IJ, MacIntyre A (2006). The association between telomere length, physical health, cognitive ageing, and mortality in non-demented older people. Neurosci Lett.

[47] Yaffe K, Lindquist K, Kluse M (2011). Telomere length and cognitive function in community-dwelling elders: findings from the Health ABC Study. Neurobiol Aging.

[48] Risques RA, Arbeev KG, Yashin AI (2010). Leukocyte telomere length is associated with disability in older u.s. Population. J Am Geriatr Soc.

[49] Collerton J, Martin-Ruiz C, Davies K (2012). Frailty and the role of inflammation, immunosenescence and cellular ageing in the very old: cross-sectional findings from the Newcastle 85+ Study. Mech Ageing Dev.

[50] Rincon M, Rudin E, Barzilai N (2005). The insulin/IGF-1 signaling in mammals and its relevance to human longevity. Exp Gerontol.

[51] de Saint-Hubert M, Jamart J, Morrhaye G (2011). Serum IL-6 and IGF-1 improve clinical prediction of functional decline after hospitalization in older patients. Aging Clin Exp Res.

