# Supplementary material for: Biological ageing and frailty markers in breast cancer patients
Source: Aging (Albany NY). 2015 May 15;7(5):319–31. doi: 10.18632/aging.100745 (PMC4468313; doi:10.18632/aging.100745)
Supplement: Supplementary file 1 [file aging-07-0319-s001.pdf]

SUPPLEMENTARY INFORMATION

Supplementary Table 1. Leuven Oncogeriatric Frailty score

Geriatric test	LOFS +2	LOFS +1	LOFS +0
ADL	6	5 - 4	≤ 3
iADL	8	7 - 4	≤ 3
MMSE	30 - 28	27 - 24	≤ 23
MNA	14 - 12	11 - 8	≤ 7
CCI	0	1	≥ 2

Supplementary Table 2. Spearman correlations between biomarkers and continuous geriatric assessment scores in the old cohort

GA scores	Telomeres			IGF-1			MCP-1			RANTES		
	N	r_s	p	N	r_s	p	N	r_s	p	N	r_s	p
LOFS	109	0.048	0.6174	114	0.124	0.1897	130	-0.017	0.8463	130	-0.025	0.7799
ECOG	120	-0.068	0.4616	127	-0.094	0.2937	150	-0.026	0.7559	150	0.031	0.7108
TRST	120	0.041	0.6604	132	-0.016	0.8537	157	0.001	0.9927	157	-0.066	0.4111
G8	120	-0.015	0.8740	118	0.041	0.6628	139	-0.112	0.1894	139	0.010	0.9066
ADL24	120	0.092	0.3162	123	-0.139	0.1243	146	0.002	0.9770	146	0.111	0.1815
IADL8	108	0.056	0.5652	123	0.073	0.4211	141	0.085	0.3153	141	0.007	0.9326
MMSE	120	0.088	0.3380	130	0.071	0.4192	153	0.153	0.0596	153	-0.058	0.4781
GDS_15	120	0.131	0.1542	130	-0.026	0.7653	153	-0.146	0.0719	153	0.015	0.8530
MNA14	120	-0.054	0.5559	118	0.096	0.2995	139	-0.053	0.5327	139	0.037	0.6662
MNA30	56	0.040	0.7681	51	0.089	0.5327	66	-0.089	0.4763	66	0.029	0.8162
CCI	120	0.009	0.9182	133	-0.195	0.0248	158	0.123	0.1244	158	-0.055	0.4905

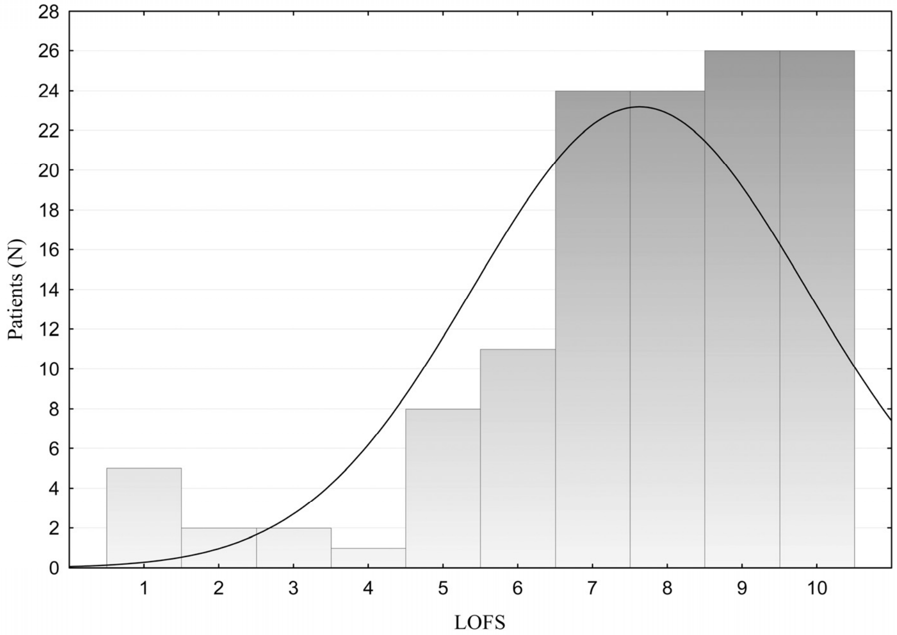

Supplementary Figure 1. LOFS distribution among older patients.

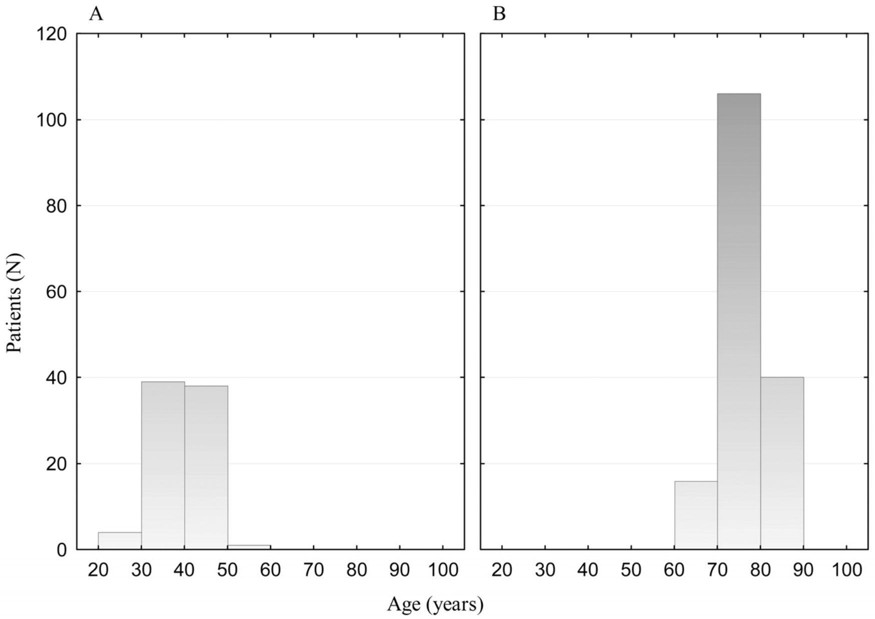

Supplementary Figure 2. Histogram of the age distribution of the study population. (**A**) young patients group (median age = 40 years, IQR = 37 – 44). (**B**) older patients group (median age = 76 years, IQR = 72 – 80).
